# Dietary intake of selected B vitamins in relation to risk of major cancers in women

**DOI:** 10.1038/sj.bjc.6604540

**Published:** 2008-07-29

**Authors:** G C Kabat, A B Miller, M Jain, T E Rohan

**Affiliations:** 1 Department of Epidemiology and Population Health, Albert Einstein College of Medicine, 1300 Morris Park Avenue, Room 1301, NY 10461, USA; 2 Department of Public Health Sciences, University of Toronto, Toronto, Canada

**Keywords:** dietary folate, thiamin, riboflavin, niacin, methionine, female neoplasms

## Abstract

Although folic acid has been investigated for its potential to inhibit carcinogenesis, few epidemiologic studies have assessed the effects of intake of thiamin, riboflavin, and niacin, which may reduce cancer risk by acting as cofactors in folate metabolism or by other mechanisms. Using data from a large cohort of Canadian women, we examined the association of dietary intake of these nutrients, as well as intake of folate, methionine, and alcohol, with cancers of the breast, endometrium, ovary, colorectum, and lung ascertained during an average of 16.4 years of follow-up. After exclusions, the following numbers of incident cases were available for analysis: breast, *n*=2491; endometrium, *n*=426; ovary, *n*=264; colorectum, *n*=617; and lung, *n*=358. Cox proportional hazard models were used to estimate risk of each cancer with individual nutrients and to explore possible effect modification by combinations of nutrients on cancer risk. Few significant associations of intake of individual B vitamins with the five cancers were observed. Alcohol consumption showed a modest positive association with breast cancer risk but not with risk of the other cancers. There was no evidence of effect modification among the nutrients. This large study provides little support for an association of dietary intake thiamin, riboflavin, niacin, folate, or methionine with five major cancers in women.

Interest in the potential of folate to reduce the risk of common cancers stems from the important role of this B vitamin in one-carbon metabolism. Optimal one-carbon metabolism is necessary for the synthesis of the purines, adenine and guanine, and the conversion of uridylate to thymidylate, which blocks the misincorporation of uracil into DNA (Choi and Mason, 2000; Ziegler and Lim, 2007). Dysregulation of one-carbon metabolism and DNA methylation is believed to promote carcinogenesis (Kim, 2004). Other B vitamins, including thiamin (vitamin B1), riboflavin (vitamin B2), and niacin (vitamin B3) may also play a role in carcinogenesis, either by acting as cofactors in folate metabolism (riboflavin (Powers, 2003, 2005)) or by their independent roles in DNA synthesis (thiamin (Boros, 2000)), maintaining genomic stability, DNA repair, and regulation of cell division and apoptosis (niacin (Jacobson *et al*, 1995; Ames, 2001; Kirkland, 2003)).

Epidemiological evidence regarding the association of folate and cancer of various sites has been inconsistent (Lewis *et al*, 2006; Larsson *et al*, 2007; Ziegler and Lim, 2007). Furthermore, few studies have assessed the roles of thiamin, riboflavin, or niacin in relation to the risk of commonly occurring cancers (Jedrychowski *et al*, 2002; van den Donk *et al*, 2005; Xu *et al*, 2007).

Given the conflicting data on folate and the paucity of studies on the other related nutrients, we examined the associations between dietary intake of folate, thiamin, riboflavin, and niacin and risk of cancers of the breast, endometrium, ovary, colorectum, and lung in the National Breast Screening Study (NBSS), a large cohort of Canadian women. In addition, we assessed the effects of intake of methionine, in view of its role as a methyl donor, and of alcohol intake, as alcohol is a known folate antagonist and may increase the requirement for folate and possibly other B vitamins (Giovannucci, 2004; Pöschl *et al*, 2004). Previous reports using the NBSS have examined the association of folate, methionine, and alcohol intake in the development of breast and colorectal cancer (Rohan *et al*, 2000; Terry *et al*, 2002a) and of folate and alcohol intake in relation to endometrial cancer (Jain *et al*, 2000b). The present study updates these earlier analyses with an additional 6 years of follow-up as well as presenting results on thiamin, riboflavin, and niacin. The five cancer sites were selected because they are among the most common cancers in women, accounting for approximately 61% of all cancers in women (Jemal *et al*, 2008).

## Materials and methods

### Study population and questionnaire

The Canadian National Breast Screening Study is a randomised controlled trial of screening for breast cancer, which has been described in detail elsewhere (Miller *et al*, 1992; Terry *et al*, 2002b). In brief, 89 835 women aged 40–59 were recruited from the general Canadian population between 1980 and 1985. On enrollment into the study, information was obtained from participants on demographic, hormonal, and reproductive characteristics using a self-administered lifestyle questionnaire. Starting in 1982, a self-administered food frequency questionnaire (FFQ) developed for the NBSS (Jain *et al*, 1982) was distributed to all new attendees at all screening centres and to women returning to the screening centres for re-screening. A total of 49 654 dietary questionnaires were returned and were available for analysis. Use of the self-administered questionnaire was validated by comparison with an interviewer-administered version of the same questionnaire (Jain *et al*, 1982). The FFQ elicited information on usual portion size and consumption of 86 food items, including alcoholic beverages, and included photographs of portion sizes to assist respondents in quantifying intake. Responses were used to estimate the daily intake of calories, alcohol, and selected nutrients with the use of a nutrient database developed by modifying the US Department of Agriculture's food composition tables to include typically Canadian foods (Jain *et al*, 1996). The estimates of nutrient intake that are the focus of this report were derived from dietary sources alone.

### Ascertainment of index cancers and deaths

Incident cases of breast, endometrial, ovarian, colorectal, and lung cancer and deaths from all causes were ascertained respectively by means of computerised record linkages to the Canadian Cancer Database and to the National Mortality Database. The linkages to the databases yielded data on cancer incidence and mortality to 31 December 2000 for women in Ontario, 31 December 1998 for women in Quebec, and 31 December 1999 for women in other provinces. For the present analyses, study participants were considered at risk from their date of enrollment until the date of diagnosis of their breast, endometrial, ovarian, colorectal, or lung cancer, termination of follow-up (the date to which cancer incidence data were available for women in the corresponding province) or death, whichever occurred first. After exclusions, during an average of 16.4 years (786 588 person-years) of follow-up of the dietary cohort, the following numbers of incident cases were available for analysis: breast, *n*=2491; endometrium, *n*=426; ovary, *n*=264; colorectum, *n*=617; and lung, *n*=358.

### Statistical analysis

Separate analyses were carried out for each cancer site. For analyses of endometrial cancer, 14 906 women who had had a hysterectomy were excluded (67 cases and 14 839 noncases). For analysis of ovarian cancer, 318 women who reported a bilateral oopherectomy were excluded (three cases and 315 noncases). In addition, for all analyses, women whose calorie intake was <730 or >5389 kcal day^−1^ (i.e., more than three s.d. from the mean of the log-transformed values) or whose body mass index (BMI) was <15 or >50 kg m^−2^ were excluded.

Cox proportional hazard models were used to estimate hazard ratios (HR) and 95% confidence intervals (CI) for the association between intake of B vitamins, methionine, and alcohol, and cancer risk. All dietary variables were adjusted for total energy intake, using the residuals method (Willett and Stampfer, 1986). For these analyses, the B vitamins of interest and methionine were categorised by quintiles based on their distribution in the total population. For alcohol, intake was categorised as nondrinker, >0 to <5, 5 to <10, 10 to <20, 20 to <30, 30+ g day^−1^. Variables were included in the multivariate models if they were established risk factors for a given cancer or if their inclusion in the model changed the parameter estimate for the nutrients of interest by more than 10%. The covariates included in the main models for the five cancers are listed in the footnotes to Table 2. Addition of indicator variables for screening centre and randomisation group in the original clinical trial (intervention/usual care) did not affect the risk estimates, and therefore these variables were omitted. To test for trends in risk with increasing levels of an exposure of interest, we assigned the median value for each quintile of that exposure variable and then fitted the medians as a continuous variable in the risk models. We then evaluated the statistical significance of the corresponding coefficient using the Wald test (Rothman and Greenland, 1998). As early symptoms of the disease might result in dietary change, we repeated the analyses for the five cancer sites, excluding cases diagnosed during the first 5 years of follow-up.

We further examined potential effect modification between nutrients by creating combined variables for each combination of two nutrients categorised into tertiles relative to the reference group containing women in the lowest tertile of both nutrients (HR=1.0). For example, women with low folate/moderate thiamine, moderate folate/moderate thiamin, high folate/moderate thiamin, and so on, were compared with women with low folate/low thiamin. Cutpoints for tertiles were: alcohol (nondrinker; >0 but <6.6 g day^−1^ (the median intake among drinkers), ⩾6.6 g day^−1^); folate (<267, 267–334, ⩾334 *μ*g day^−1^); thiamin (<1.15, 1.15 to <1.33, ⩾1.33 mg day^−1^); riboflavin (<1.56, 1.56 to <1.93, ⩾1.93 mg day^−1^); niacin (<18.1, 18.1 to <21.0, ⩾21.0 mg day^−1^); and methionine (<1.92, 1.92 to <2.26, ⩾2.26 g day^−1^). We examined the statistical significance of the HRs in the resulting 3 × 3 tables. Tests for interaction were based on the likelihood ratio tests comparing models with and without the product terms representing the variables of interest as continuous variables. The likelihood ratio test that all of the interaction parameters were 0 was performed by referring 2^*^ the absolute difference in the log likelihoods of the models with and without the interaction terms to the *χ*^2^ distribution with 1 degree of freedom. All statistical significance tests were two-sided. All analyses were performed using SAS version 9 (SAS Institute Cary, NC, USA).

## Results

Correlations between the B vitamins, methionine, and alcohol are shown in Table 1. Intakes of niacin and methionine showed the strongest correlation (*r*=0.56), followed by folate and thiamin (*r*=0.49), riboflavin and methionine (*r*=0.47), and intake of folate and riboflavin (*n*=0.43). Intakes of B vitamins and methionine were inversely but weakly correlated with alcohol consumption.

### Breast cancer

None of the B vitamins or methionine was associated with risk of breast cancer, whereas alcohol intake showed a weak, borderline positive association: the HR for those consuming 30+ g of alcohol per day was 1.17, 95% CI 0.98–1.39 (Table 2). Results were similar in analyses stratified by menopausal status (premenopausal, postmenopausal) and were not affected by exclusion of cases diagnosed during the first 5 years of follow-up (data not shown). There was no evidence of effect modification when combinations of nutrients and alcohol were considered, and none of the formal tests for interaction was statistically significant.

### Endometrial cancer

None of the B vitamins, methionine, or alcohol was associated with risk of endometrial cancer (Table 2). The results were not affected by excluding cases diagnosed during the first 5 years of follow-up. There were no significant interactions between nutrients or between nutrients and alcohol and endometrial cancer risk.

### Ovarian cancer

The highest quintile of riboflavin intake was associated with reduced risk: HR 0.57, 95% CI 0.37–0.86, *P*=0.05 (Table 2). The association was unchanged when cases diagnosed during the first 5 years of follow-up were excluded. There were no other significant associations with ovarian cancer and no interactions between nutrients and ovarian cancer.

### Colorectal cancer

The B vitamins, methionine, and alcohol were not associated with colorectal cancer risk (Table 2) or with subsites within the colorectum (proximal colon, distal colon, and rectum) (data not shown). When cases diagnosed during the first 5 years of follow-up were excluded, thiamin intake showed an inverse association with colorectal cancer risk (HR for highest *vs* lowest quintile 0.78, 95% CI 0.59–1.04, *P* for trend 0.02). There were no significant interactions between nutrients, or between nutrients and alcohol intake, and colorectal cancer risk.

### Lung cancer

Riboflavin intake showed a borderline positive association with lung cancer risk: HR for highest quintile of intake 1.30, 95% CI 0.94–1.80, *P*=0.08 (Table 2). When cases diagnosed during the first 5 years of follow-up were excluded, the HR was 1.38, 95% CI 0.98–1.94, *P*=0.04. There were no interactions between different nutrients and lung cancer risk.

## Discussion

Results from this large cohort of women followed for an average of 16 years provide little support for an association of folate, thiamin, riboflavin, niacin, or methionine and risk of five major cancers in women. Alcohol consumption showed a weak positive association with breast cancer but not with any of the other cancer sites. There was no suggestion of an interaction between alcohol consumption and intake of individual micronutrients or between any of the micronutrients with each other in relation to risk of cancer at the five anatomic sites.

Our null findings regarding folate and breast cancer are in agreement with the results of two recent meta-analyses (Lewis *et al*, 2006; Larsson *et al*, 2007), as well as with results from an earlier analysis of the NBSS (Rohan *et al*, 2000), and our results concerning folate and endometrial cancer are in agreement with those of a previous report from the NBSS cohort (Jain *et al*, 2000a). A recent meta-analysis of the relationship between folate intake and colorectal cancer risk (Sanjoaquin *et al*, 2005) showed a modest inverse association with dietary intake (RR for high *vs* low intake 0.75, 95% CI 0.64–0.89) but not with total folate intake (RR 0.95, 95% CI 0.81–1.11); however, a large study of folate intake and colorectal adenoma (Flood *et al*, 2002) found no association. Results of cohort studies of the association between folate and ovarian cancer are inconsistent (Kelemen *et al*, 2004; Larsson *et al*, 2004; Tworoger *et al*, 2006). Our results for lung cancer are consistent with those from a pooled analysis of eight prospective studies (Cho *et al*, 2006) but contrast with those of a cohort study from the Netherlands (Voorrips *et al*, 2000) showing an inverse association between folate intake and lung cancer, which was strongest in current smokers.

Findings regarding the association of methionine with cancers of the breast, endometrium, and ovary have generally been null (Thorand *et al*, 1998; Shrubsole *et al*, 2001; Feigelson *et al*, 2003; Tworoger *et al*, 2006; Xu *et al*, 2007; Cho *et al*, 2007a), whereas for colorectal cancer, the findings have been mixed, with some suggesting an inverse association (Giovannucci *et al*, 1993, 1995, 1998) and others showing no association (Ferraroni *et al*, 1994; Slattery *et al*, 1997; Flood *et al*, 2002; Harnack *et al*, 2002). We are unaware of any previous studies that have examined the association of methionine intake and lung cancer risk.

Few studies have examined the association of thiamin, riboflavin, or niacin with common epithelial cancers. One small case–control study of colorectal cancer (Jedrychowski *et al*, 2002) observed a significant inverse association with dietary thiamin intake but not with riboflavin intake. A second case–control study (Ferraroni *et al*, 1994) reported a significant inverse association of thiamin intake with colon cancer risk in both males and females, but found no association with niacin or folate intake. In a large case–control study from China (Xu *et al*, 2007), intake of riboflavin was not associated with endometrial cancer risk. To our knowledge, no cohort studies have reported on the association of thiamin, riboflavin, or niacin with any of the cancers addressed here.

Our findings of a significant inverse association for the highest *vs* the lowest quintile of riboflavin intake with ovarian cancer, a borderline inverse association of thiamin intake with colorectal cancer, and a borderline positive association of riboflavin intake with lung cancer risk should be interpreted cautiously, considering the modest nature of these associations and the large number of comparisons.

In all dietary studies focusing on individual nutrients, there is the potential for confounding due to the fact that many nutrients are correlated (either positively or inversely) with each other (Key, 1994; Bingham, 2006). Furthermore, the levels and activity of one nutrient may be affected by those of other nutrients in the same pathway or interacting pathways. One-carbon metabolism may involve as many as 25 enzymes, some of which require the presence of vitamin B-6, vitamin B-12, and riboflavin, in addition to folate, as coenzymes (Ziegler and Lim, 2007). Availability of these nutrients is influenced by diet, use of supplements, alcohol consumption, and genetic polymorphisms that play a role in the metabolism of folate and other B vitamins. Furthermore, there is suggestive evidence that, depending on the dose and timing of administration, supplemental folate may have adverse effects on colorectal and ovarian cancer risk (Kelemen *et al*, 2004; Cole *et al*, 2007; Ziegler and Lim, 2007). In view of this complexity and the problems of using estimates of intake of individual nutrients obtained at one point in time, the potential role of B vitamins in providing protection against common cancers remains unresolved.

Strengths of the present study include large numbers of the five cancers, the high level of completeness of follow-up of the cohort, and the availability of baseline information on a wide range of lifestyle exposures. In the case of breast cancer particularly, the large sample size afforded good statistical power to detect associations in subgroups and interactions. Limitations include the fact that we only had exposure information obtained at baseline and that dietary patterns, and hence intake of specific nutrients, may have changed over the long follow-up period, leading to misclassification of exposure and reduced power to detect associations. Another limitation is that the FFQ, with 86 food items, may have omitted foods that make a substantial contribution to the nutrients that we focused on. Furthermore, information on use of these vitamins as supplements was not obtained in this study. Finally, intake of other vitamins and nutrients, such as vitamin B-6, vitamin B-12, choline, and betaine, which play a role in one-carbon metabolism and may influence cancer risk (Ziegler and Lim, 2007; Cho *et al*, 2007b; Xu *et al*, 2008), was not computed in the NBSS nutrient data base.

In conclusion, the present study provides little evidence for associations between dietary intake of folate, thiamin, riboflavin, niacin, or methionine and risk of cancers of the breast, endometrium, ovary, colorectum, or lung. However, given the potential role of these vitamins and nutrients in carcinogenesis and particularly given the paucity of studies to date regarding intake of thiamin, riboflavin, and niacin and the risk of epithelial cancers, further investigations are warranted.

## Figures and Tables

**Table 1:** Pearson correlation coefficients for intake of B vitamins and other factors among 49 654 women in the NBSS^a^

	**Folate**	**Thiamine**	**Riboflavin**	**Niacin**	**Methionine**
Folate					
Thiamin	0.49				
Riboflavin	0.43	0.32			
Niacin	0.20	0.27	0.13		
Methionine	0.12	0.18	0.47	0.56	
Alcohol	−0.05	−0.18	−0.13	−0.15	−0.15

a All correlations are significant at *P*<0.0001.

**Table 2:** Multivariable-adjusted hazard ratios and 95% confidence intervals for the association of dietary intake of B-vitamins and other factors with risk of breast, endometrial, ovarian, colorectal, and lung cancer in the NBSS

**Characteristic quintiles**	**Breast cancer (*n* cases=2491) HR^a^ (95% CI)**	**Endometrial cancer (*n* cases=426) HR^b^ (95% CI)**	**Ovarian cancer (*n*=264) HR^c^ (95% CI)**	**Colorectal cancer (*n* cases=617) HR^d^ (95% CI)**	**Lung cancer quintiles (*n*=358) HR^e^ (95% CI)**
*Folate (μg day*^*−1*^)					
<237	1.00 (reference)	1.00 (reference)	1.00 (reference)	1.00 (reference)	1.00 (reference)
237 to <281	0.99 (0.87–1.13)	0.91 (0.65–1.26)	1.13 (0.78–1.64)	0.99 (0.76–1.29)	0.81 (0.59–1.11)
281 to <321	1.03 (0.91–1.17)	1.07 (0.78–1.48)	0.91 (0.62–1.35)	1.09 (0.84–1.41)	0.64 (0.45–0.91)
321 to <374	0.98 (0.86–1.12)	1.12 (0.81–1.54)	1.09 (0.74–1.59)	1.12 (0.86–1.44)	0.93 (0.67–1.28)
374+	1.02 (0.90–1.17)	0.79 (0.55–1.13)	1.05 (0.71–1.54)	0.89 (0.68–1.17)	1.12 (0.83–1.52)
*P trend*	*0.79*	*0.55*	*0.92*	*0.74*	*0.43*
					
*Thiamin (mg day*^*−1*^)					
<1.06	1.00 (reference)	1.00 (reference)	1.00 (reference)	1.00 (reference)	1.00 (reference)
1.06 to <1.19	1.08 (0.95–1.23)	1.34 (0.96–1.86)	0.91 (0.63–1.31)	1.06 (0.82–1.36)	0.96 (0.69–1.32)
1.19 to <1.30	1.11 (0.98–1.27)	1.19 (0.85–1.68)	0.83 (0.57–1.21)	1.12 (0.87–1.45)	1.03 (0.75–1.42)
1.30 to <1.43	1.05 (0.93–1.20)	1.26 (0.90–1.77)	0.88 (0.61–1.28)	0.90 (0.69–1.17)	1.13 (0.82–1.56)
1.43+	1.04 (0.91–1.18)	1.27 (0.90–1.78)	0.78 (0.53–1.15)	0.82 (0.62–1.08)	0.99 (0.71–1.38)
*P trend*	*0.79*	*0.35*	*0.22*	*0.07*	*0.69*
					
*Riboflavin (mg day*^*−1*^)					
<1.41	1.00 (reference)	1.00 (reference)	1.00 (reference)	1.00 (reference)	1.00 (reference)
1.41 to <1.63	0.98 (0.86–1.11)	0.86 (0.61–1.21)	0.83 (0.57–1.20)	1.03 (0.80–1.33)	1.06 (0.76–1.46)
1.63 to <1.86	0.96 (0.84–1.09)	0.94 (0.68–1.32)	0.90 (0.63–1.30)	0.99 (0.77–1.28)	1.08 (0.77–1.51)
1.86 to <2.17	0.98 (0.87–1.12)	0.79 (0.55–1.13)	0.95 (0.66–1.36)	0.85 (0.65–1.11)	1.20 (0.86–1.67)
2.17+	0.99 (0.87–1.12)	0.78 (0.52–1.17)	0.57 (0.37–0.86)	0.94 (0.73–1.23)	1.30 (0.94–1.80)
*P trend*	*0.89*	*0.22*	*0.05*	*0.31*	*0.08*
					
*Niacin (mg day*^*−1*^)					
<16.9	1.00 (reference)	1.00 (reference)	1.00 (reference)	1.00 (reference)	1.00 (reference)
16.9 to <18.7	0.96 (0.84–1.09)	1.08 (0.79–1.47)	1.09 (0.75–1.58)	1.08 (0.84–1.39)	1.14 (0.80–1.62)
18.7 to <20.5	0.98 (0.86–1.11)	1.05 (0.77–1.44)	0.89 (0.60–1.32)	1.08 (0.85–1.39)	0.95 (0.66–1.35)
20.5 to <22.8	0.97 (0.85–1.10)	0.93 (0.67–1.28)	1.05 (0.72–1.53)	0.97 (0.75–1.25)	1.03 (0.72–1.45)
22.8+	0.97 (0.85–1.10)	0.90 (0.64–1.26)	0.98 (0.66–1.45)	0.95 (0.74–1.23)	1.19 (0.86–1.65)
*P trend*	*0.74*	*0.35*	*0.87*	*0.43*	*0.43*
					
*Methionine (g day*^*−1*^)					
<1.78	1.00 (reference)	1.00 (reference)	1.00 (reference)	1.00 (reference)	1.00 (reference)
1.78 to <2.00	1.04 (0.92–1.19)	0.90 (0.64–1.25)	1.05 (0.73–1.51)	0.99 (0.77–1.28)	0.80 (0.58–1.10)
2.00 to <2.20	1.08 (0.95–1.22)	1.04 (0.76–1.43)	0.80 (0.55–1.17)	0.91 (0.71–1.18)	0.73 (0.52–1.03)
2.20 to <2.48	0.98 (0.86–1.12)	0.93 (0.67–1.28)	0.94 (0.64–1.37)	0.92 (0.71–1.20)	1.05 (0.77–1.42)
2.48+	1.00 (0.88–1.14)	0.77 (0.54–1.08)	0.89 (0.61–1.31)	0.99 (0.76–1.28)	0.95 (0.69–1.31)
*P trend*	*0.68*	*0.20*	*0.42*	*0.73*	*0.76*
					
*Alcohol*					
Nondrinker	1.00 (reference)	1.00 (reference)	1.00 (reference)	1.00 (reference)	1.00 (reference)
>0 to <5 g day^−1^	1.00 (0.90–1.12)	1.15 (0.88–1.51)	0.90 (0.63–1.29)	0.92 (0.73–1.14)	0.94 (0.69–1.27)
5 to <10 g day^−1^	0.98 (0.86–1.13)	1.00 (0.71–1.41)	1.33 (0.89–1.96)	0.93 (0.71–1.22)	0.77 (0.52–1.13)
10 to <20 g day^−1^	1.07 (0.93–1.23)	1.21 (0.86–1.68)	0.85 (0.54–1.32)	1.04 (0.79–1.36)	0.76 (0.53–1.11)
20 to <30 g day^−1^	1.08 (0.89–1.32)	1.34 (0.85–2.12)	1.50 (0.88–2.56)	1.13 (0.77–1.64)	0.81 (0.49–1.34)
30+	1.17 (0.98–1.39)	0.84 (0.52–1.36)	1.23 (0.74–2.04)	1.02 (0.72–1.44)	1.03 (0.71–1.51)
*P trend*	*0.06*	*0.04*	*0.22*	*0.48*	*0.12*

a Adjusted for age (continuous), body mass index (kg/m^2^ – continuous), pack-years of smoking (none, >0 to <10, 10 to <20, 20 to <30, 30+), years of education (three levels), menopausal status (pre-, peri-, post), family history of breast cancer (no, yes), history of breast biopsy (no, yes), age at menarche (<12, 12, 13, >13), parity (continuous), oral contraceptive use (never, ever), hormone replacement therapy (never, ever), and intake of calories (continuous). In addition, all nutrients except alcohol were adjusted for alcohol intake (continuous).

b Adjusted for age (continuous), body mass index (kg/m^2^ – continuous), years of education (three levels), menopausal status (pre-, peri-, post), parity (continuous), age at menarche (<12, 12, 13, >13), oral contraceptive use (ever, never), hormone replacement therapy (never, ever), and intake of calories, calcium, and raw vegetables (all continuous). In addition, all nutrients except alcohol were adjusted for alcohol intake (continuous).

c Adjusted for age (continuous), body mass index (kg/m^2^ – continuous), years of education (three levels), menopausal status (pre-, peri-, post), parity (continuous), duration of oral contraceptive use (4 levels), duration of hormone replacement use (4 levels), hysterectomy (yes/no).

d Adjusted for age (continuous), body mass index (kg/m^2^ – continuous), pack-years of smoking (none, >0 to <10, 10 to <20, 20 to <30, 30+), years of education (3 levels), menopausal status (pre-, peri-, post), oral contraceptive use (never, ever), hormone replacement therapy (never, ever), and intake of calories (continuous). In addition, all nutrients except alcohol were adjusted for alcohol intake (continuous).

e Adjusted for age (continuous), body mass index (kg/m^2^ – continuous), years of education (three levels), parous/nulliparous, smoking status, pack-years of smoking (Never, 1–9, 10–19, 20–29, 30–39, 40+), and alcohol (continuous).
